# SARS-CoV-2 Distribution in Residential Housing Suggests Contact Deposition and Correlates with *Rothia* sp.

**DOI:** 10.1128/msystems.01411-21

**Published:** 2022-05-16

**Authors:** Victor J. Cantú, Rodolfo A. Salido, Shi Huang, Gibraan Rahman, Rebecca Tsai, Holly Valentine, Celestine G. Magallanes, Stefan Aigner, Nathan A. Baer, Tom Barber, Pedro Belda-Ferre, Maryann Betty, MacKenzie Bryant, Martín Casas Maya, Anelizze Castro-Martínez, Marisol Chacón, Willi Cheung, Evelyn S. Crescini, Peter De Hoff, Emily Eisner, Sawyer Farmer, Abbas Hakim, Laura Kohn, Alma L. Lastrella, Elijah S. Lawrence, Sydney C. Morgan, Toan T. Ngo, Alhakam Nouri, Ashley Plascencia, Christopher A. Ruiz, Shashank Sathe, Phoebe Seaver, Tara Shwartz, Elizabeth W. Smoot, R. Tyler Ostrander, Thomas Valles, Gene W. Yeo, Louise C. Laurent, Rebecca Fielding-Miller, Rob Knight

**Affiliations:** a Department of Bioengineering, University of California San Diego, La Jolla, CA, USA; b Department of Pediatrics, University of California San Diego, La Jolla, CA, USA; c Bioinformatics and Systems Biology Graduate Program, University of California San Diego, La Jolla, CA, USA; d Department of Obstetrics, Gynecology, and Reproductive Sciences, University of California San Diego, La Jolla, CA, USA; e Sanford Consortium of Regenerative Medicine, University of California San Diego, La Jolla, CA, USA; f Expedited COVID Identification Environment (EXCITE) Laboratory, Department of Pediatrics, University of California San Diego, La Jolla, CA, USA; g Department of Cellular and Molecular Medicine, University of California San Diego, La Jolla, CA, USA; h Rady Children's Hospital, San Diego, CA, USA; i San Diego State University, San Diego, CA, USA; j Department of Obstetrics, Gynecology, and Reproductive Sciences, University of California San Diego, La Jolla, CA, USA; k Herbert Wertheim School of Public Health, University of California San Diego, La Jolla, CA, USA; l Department of Computer Science and Engineering, University of California San Diego, La Jolla, CA, USA; m Center for Microbiome Innovation, University of California San Diego, La Jolla, CA, USA; Princeton University

**Keywords:** COVID-19, RT-qPCR, Rothia, SARS-CoV-2, built-environment, environmental monitoring, isolation, quarantine, surface sampling, swab

## Abstract

Monitoring severe acute respiratory syndrome coronavirus 2 (SARS-CoV-2) on surfaces is emerging as an important tool for identifying past exposure to individuals shedding viral RNA. Our past work demonstrated that SARS-CoV-2 reverse transcription-quantitative PCR (RT-qPCR) signals from surfaces can identify when infected individuals have touched surfaces and when they have been present in hospital rooms or schools. However, the sensitivity and specificity of surface sampling as a method for detecting the presence of a SARS-CoV-2 positive individual, as well as guidance about where to sample, has not been established. To address these questions and to test whether our past observations linking SARS-CoV-2 abundance to *Rothia* sp. in hospitals also hold in a residential setting, we performed a detailed spatial sampling of three isolation housing units, assessing each sample for SARS-CoV-2 abundance by RT-qPCR, linking the results to 16S rRNA gene amplicon sequences (to assess the bacterial community at each location), and to the Cq value of the contemporaneous clinical test. Our results showed that the highest SARS-CoV-2 load in this setting is on touched surfaces, such as light switches and faucets, but a detectable signal was present in many untouched surfaces (e.g., floors) that may be more relevant in settings, such as schools where mask-wearing is enforced. As in past studies, the bacterial community predicts which samples are positive for SARS-CoV-2, with *Rothia* sp. showing a positive association.

**IMPORTANCE** Surface sampling for detecting SARS-CoV-2, the virus that causes coronavirus disease 2019 (COVID-19), is increasingly being used to locate infected individuals. We tested which indoor surfaces had high versus low viral loads by collecting 381 samples from three residential units where infected individuals resided, and interpreted the results in terms of whether SARS-CoV-2 was likely transmitted directly (e.g., touching a light switch) or indirectly (e.g., by droplets or aerosols settling). We found the highest loads where the subject touched the surface directly, although enough virus was detected on indirectly contacted surfaces to make such locations useful for sampling (e.g., in schools, where students did not touch the light switches and also wore masks such that they had no opportunity to touch their face and then the object). We also documented links between the bacteria present in a sample and the SARS-CoV-2 virus, consistent with earlier studies.

## OBSERVATION

Environmental monitoring for severe acute respiratory syndrome coronavirus 2 (SARS-CoV-2) RNA by reverse transcription-quantitative PCR (RT-qPCR) is increasingly gaining acceptance. In the Safer at School Early Alert (SASEA) (https://saseasystem.org/) project, daily surface swabbing was employed as part of an effort to detect coronavirus disease 2019 (COVID-19) cases in nine elementary schools. This study identified 89 clinically positive COVID-19 cases, with 33% of the positive cases preceded by a room-matched surface positive (1). As pandemic response measures like SASEA become more widely implemented, understanding where SARS-CoV-2 signatures will most likely be found reduces the cost and labor of surface swabbing in large facilities. Previous work focused on sampling arbitrary surfaces in isolation and congregate-care facilities, homes, and hospitals, with various detection performances obscuring which surfaces are best for monitoring COVID-19 spread (2–6). Counterintuitively, high-touch hospital surfaces expected to accumulate viral load, including door handles and patient bed rails, can yield lower SARS-CoV-2 detection rates, presumably because they are cleaned more often (7, 8).

Most microbes in the built environment come from human inhabitants (9–11). Oral, gut, and skin microbiomes of COVID-19 patients change during disease (8, 12, 13). Therefore, SARS-CoV-2 positive built environmental samples may differ in bacterial communities from SARS-CoV-2 negative samples. This has been documented in a hospital setting, with associations between SARS-CoV-2 status (detected/not detected) and both the overall microbial community and *Rothia* sp. specifically (8).

To extend these results to a residential setting and understand how SARS-CoV-2 is distributed in the living space of an infected individual, we performed environmental sampling in the apartments of three people who recently tested positive for COVID-19 (Fig. S1) while quarantined in an isolation facility. On the day of swabbing, each quarantining individual provided an anterior nares swab sample (average Cq: 29.5, 28.4, 28.6 for apartments A, B, and C, respectively). Although apartments differed in size, floor plan, and features (furniture, appliances, etc.), similar features at similar densities were swabbed across all three (*n* = 140, 116, and 125).

10.1128/msystems.01411-21.1 FIG S1 Timeline of events from the first positive test to the end of the individual’s quarantine period. Apartment C had no move-in date because the individual was quarantined in place. Download FIG S1, JPG file, 0.3 MB. Copyright © 2022 Cantú et al. 2022 Cantú et al. https://creativecommons.org/licenses/by/4.0/ This content is distributed under the terms of the Creative Commons Attribution 4.0 International license.

Each sampled surface was swabbed twice in immediately adjacent locations: first with a swab premoistened and stored in 95% ethanol, then by a second swab premoistened and stored in a 0.5% SDS wt/vol solution (Text S1). Ethanol samples underwent 16S V4 rRNA gene amplicon (16S) sequencing, and SDS samples underwent RT-qPCR for SARS-CoV-2 detection. In the 16S sequencing, sequences were demultiplexed, quality filtered, and denoised with deblurring (14) in Qiita (15) (study ID:13957) using default parameters. The resulting feature tables were processed using QIIME2 (16).

10.1128/msystems.01411-21.7 TEXT S1 Description of the experimental methods and data analysis. Download Text S1, PDF file, 0.09 MB. Copyright © 2022 Cantú et al. 2022 Cantú et al. https://creativecommons.org/licenses/by/4.0/ This content is distributed under the terms of the Creative Commons Attribution 4.0 International license.

## RESULTS

**FIG 1:**
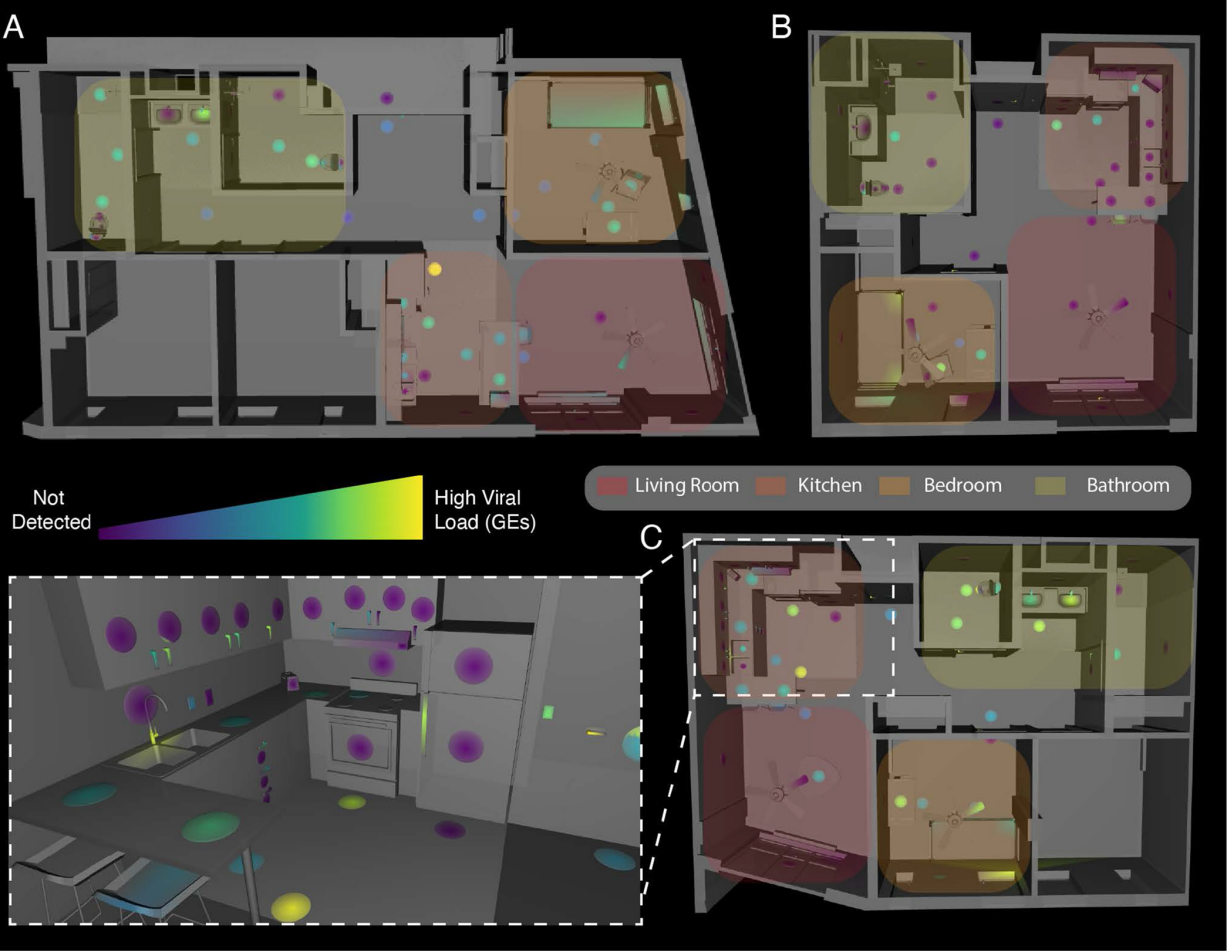
Distribution of SARS-CoV-2 viral load in isolation dorm apartments. (A to C) Floor plans for each apartment highlighted where SARS-CoV-2 RNA signatures were detected. (Inset) 3D rendering of the kitchen in apartment C showing SARS-CoV-2 viral load in genomic equivalents (GEs) mapped to features in that room.

We collected 381 matched 16S and SARS-CoV-2 surface samples from the three apartments, of which 178 (47%) were positive for SARS-CoV-2 (Fig. 1) (Table S1). Apartments A and C had comparable positivity rates (53% and 61%, respectively), but apartment B was substantially lower (24%). In all three apartments, the rate of detection was highest in the bedroom (72% on average versus 47% overall). The swabbed surfaces were grouped into three categories: high-touch, low-touch, or floors. High-touch surfaces included door handles, switches, and countertops while walls, door faces, and ceiling fans were examples of low-touch surfaces. High-touch surfaces and floors had positivity rates 2 to 3 times higher than low-touch surfaces across all apartments (Table S2).

10.1128/msystems.01411-21.5 TABLE S1 Environmental samples with detectable SARS-CoV-2 per apartment and room type. Download Table S1, XLSX file, 0.07 MB. Copyright © 2022 Cantú et al. 2022 Cantú et al. https://creativecommons.org/licenses/by/4.0/ This content is distributed under the terms of the Creative Commons Attribution 4.0 International license.

10.1128/msystems.01411-21.6 TABLE S2 Environmental samples with detectable SARS-CoV-2 per apartment and surface type. Download Table S2, XLSX file, 0.01 MB. Copyright © 2022 Cantú et al. 2022 Cantú et al. https://creativecommons.org/licenses/by/4.0/ This content is distributed under the terms of the Creative Commons Attribution 4.0 International license.

We estimated surface viral load, in viral genomic equivalents (GE’s), from Cq’s using published regression curves (17) and mapped resulting viral loads onto 3D renderings of each apartment. High-touch surfaces had the highest viral load across all apartments, followed by floor samples and then high-use objects (fridge, sinks, toilets, and beds) (Fig. 1). The maps for each apartment were studied to understand patterns of SARS-CoV-2 detection and deposition by room use. In the kitchens, objects with planar faces and handles, such as the refrigerator, cabinets, and drawers, revealed that only the touched handles had detectable RT-qPCR signal (Fig. 1C inset, as an example). We could not detect viral RNA on adjacent planar faces, which were presumably breathed on but not touched.

For quality control of 16S sequencing from low-biomass samples, we sequenced surface swabs from the apartments together with positive and negative controls using KatharoSeq (Text S1; Fig. S2A) (18). Of 381 samples that underwent 16S sequencing, 121 fell below the KatharoSeq threshold and were excluded (Fig. S2C). Informed by alpha rarefaction curves (Fig. S2B), the remaining samples were rarefied to 4000 features (suboperational-taxonomic-units [sOTUs] (14)), removing an additional 36 samples from the analysis. Therefore, 157 samples were excluded from downstream analyses (122 SARS-CoV-2 negative matched swabs, 35 positive) (Fig. S2C).

10.1128/msystems.01411-21.2 FIG S2 Exclusion criteria for low biomass samples. (A) The diluted stock of a KatharoSeq positive-control was sequenced along with the environmental samples and the resultant reads underwent preprocessing as detailed in Text S1. The KatharoSeq threshold (dashed lined), a minimum number of reads derived from a fitted allosteric sigmoidal curve, corresponded to a sequencing depth where at least 80% of the positive-control reads are taxonomically classified to the appropriate target organisms (B) Top: rarefaction curve showing observed features (alpha diversity metric) as a function of sequencing depth. Bottom: graph showing how many samples would be included in the downstream analysis as a function of rarefaction depth. (C) Table showing how many samples were removed at the KatharoSeq and Rarefaction thresholds and overall ([+] = SARS-CoV-2 positive, [−] = SARS-CoV-2 negative). Download FIG S2, TIF file, 0.9 MB. Copyright © 2022 Cantú et al. 2022 Cantú et al. https://creativecommons.org/licenses/by/4.0/ This content is distributed under the terms of the Creative Commons Attribution 4.0 International license.

Bacterial alpha diversity analysis revealed a significant difference in Faith’s phylogenetic diversity (Faith’s PD) metric between SARS-CoV-2 detection status groupings at the whole data set level but demonstrated limited significant differences within apartments or room types (Fig. S3). Forward stepwise redundancy analysis (RDA) using the unweighted UniFrac beta diversity metric identified four nonredundant variables of significant effect size (apartment, surface material, type of room, and SARS-CoV-2 detection status) which accounted for 45.4% of the variation in the data (Fig. S4B). Analyzed by apartment, only in apartment B did virus detection lack significant effect. When subsetting the entire data set by room type, detection status had a significant effect on variability across all rooms.

10.1128/msystems.01411-21.3 FIG S3 Associations between microbial diversity and SARS-CoV-2 detection. (A) Mann-Whitney U test comparing ranked Faith’s PD values from microbiome samples showed a significant difference when grouped by SARS-CoV-2 status (U = 7767; ***, *P* = 2.26 × 10^−5^). (B) When subsetting the samples by apartment, only apartment A showed a significant difference in Faith’s PD values between SARS-CoV-2 status groups (**, *P* ≤ 0.001). (C) When subsetting the samples by room type, only the bedroom and kitchen showed significant differences in Faith’s PD values between SARS-CoV-2 status groups (*, *P* ≤ 0.01). Download FIG S3, TIF file, 1.5 MB. Copyright © 2022 Cantú et al. 2022 Cantú et al. https://creativecommons.org/licenses/by/4.0/ This content is distributed under the terms of the Creative Commons Attribution 4.0 International license.

10.1128/msystems.01411-21.4 FIG S4 Beta diversity analysis identifies the factors that contribute most to the separation of the data. (A) Principal coordinates analysis of the unweighted Unifrac distance matrix showed a major driver in the separation of the data from which apartment the samples came from. (B) Bar plot showing the statistically significant effect sizes for nonredundant variables returned by RDA analysis. The largest effect size was explained by apartment (30.7%, *P* = 0.0002) followed by surface material (10.7%, *P* = 0.0002), room type (3.2%, *P* = 0.0004), and SARS-CoV-2 detection status (0.84%, *P* = 0.01). Download FIG S4, TIF file, 0.6 MB. Copyright © 2022 Cantú et al. 2022 Cantú et al. https://creativecommons.org/licenses/by/4.0/ This content is distributed under the terms of the Creative Commons Attribution 4.0 International license.

**FIG 2:**
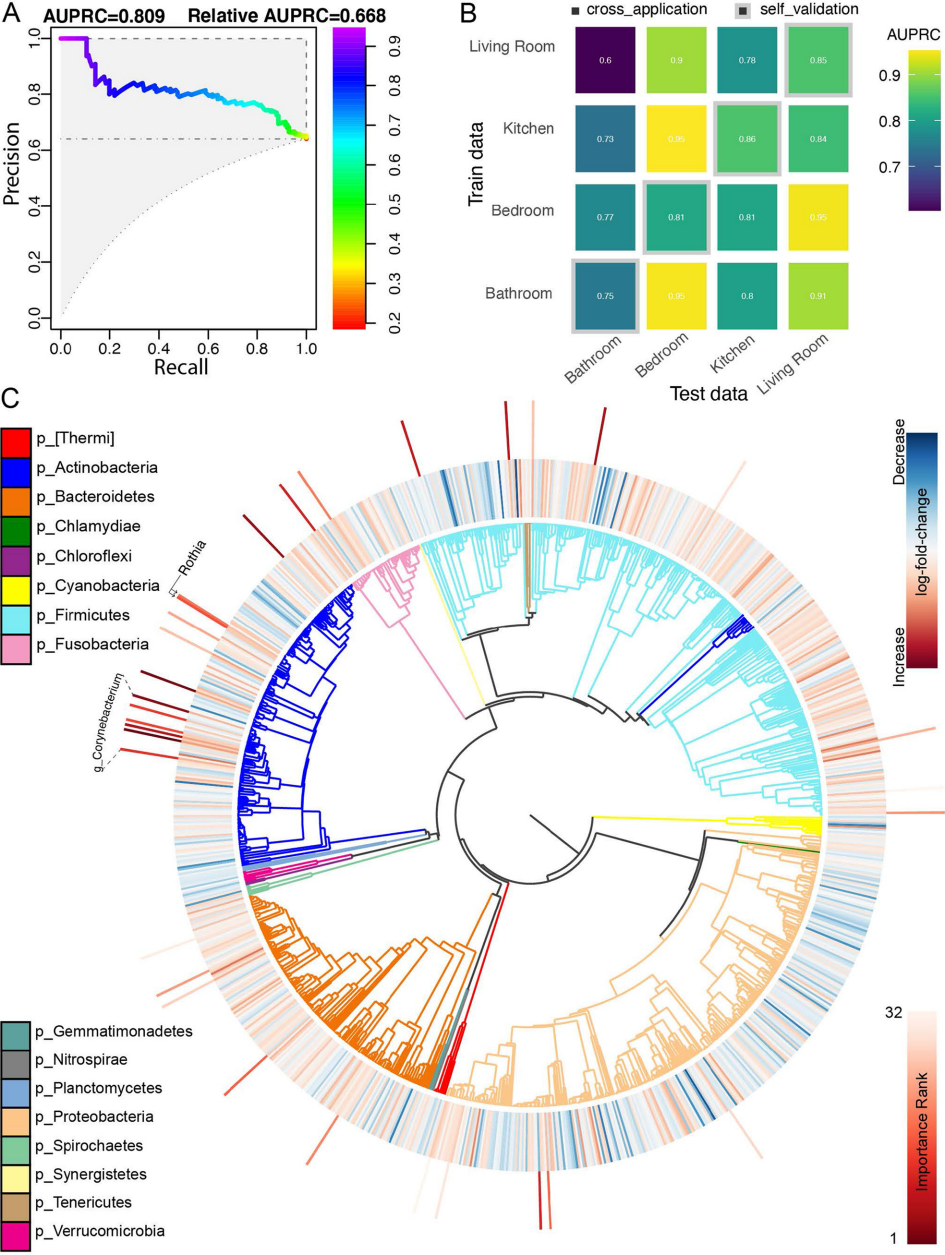
(A) Area under the precision-recall curve showing the overall prediction performance of the random forest classifiers when trained on the features from two apartments and cross-validated on the remaining apartment. (B) Confusion matrix showing per-room type classifiers’ performances (AURPC) when cross-applied to the remaining room types. The diagonal represents self-validation. (C) Phylogenetic tree visualization (EMPress) where the differentially abundant features between SARS-CoV-2 status groups identified by multinomial regression (Songbird) are plotted on the inner ring (red: positive fold change in SARS-CoV-2 positive group; blue: negative fold change in SARS-CoV-2 positive group) and the ranked sOTUs (top 32) identified as important by the random forest classifier are indicated on the outer ring. Leaves of the phylogenetic tree represent sOTUs relevant to the microbiome diversity and differential abundance analyses (number of sOTUs = 1047). The taxonomic classification (p_:phylum) of the sOTUs is indicated as colored branches in the phylogenetic tree.

To test whether the bacterial community predicted SARS-CoV-2 status, we built a random forest classifier using rarefied sOTU data. The overall area under the precision-recall curve (AUPRC) was 0.78, suggesting a statistically significant association, but insufficiently strong to predict the SARS-CoV-2 status of a single sample from the bacterial community (Fig. 2A and B). We also applied compositionally aware, multinomial regression to our data set to identify differentially abundant microbes between SARS-CoV-2 status groups (19). Because this regression model implicitly accounts for variable sequencing depth by modeling the relative fold change of each feature in centered log-ratio (CLR) coordinates (20), we used unrarefied data as an input exclusively for this method (details in Text S1). The top 32 features identified by the random forest classifier and the ranked fold changes in feature abundance from the multinomial regression are shown in Fig. 2C. Agreeing with previously published findings, Rothia dentocariosa was one of the top features identified by the classifier and was relatively positively associated with SARS-CoV-2 positive samples in the regression (8, 12). Six sOTUs belonging to members of the genus *Corynebacterium* were also highly ranked as predictive for positive samples (Fig. 2C).

Our results showed that detailed spatial mapping of SARS-CoV-2 RNA abundance and associated bacterial signatures from built environment surfaces provided useful insight into potential sampling locations and associations between the viral and bacterial components of the microbiome. In the residential setting, high-touch surfaces have especially high viral loads, although confirming this with detailed spatial maps in other settings (hospitals, isolation hotels, and schools) may be useful for guiding sampling designs. However, while high-touch surfaces have higher viral loads, floors had the highest rate of positivity, effectively rendering both floors and high-touch surfaces as good candidates for detecting SARS-CoV-2 indoors. We note that the sensitivity of arbitrary single surface sampling to detect the presence of even an unmasked COVID-19 patient was low, which was evidenced in apartment B where approximately only 1 in 4 random surface samples returned a SARS-CoV-2 detection event, so multiple samples or samples from selected surfaces should be collected. Although apartment B had a considerably lower rate of positivity, trends of SARS-CoV-2 detection across indoor spaces and surface types closely mirrored those seen in the other two apartments in this study, and largely agree with other surveys of SARS-CoV-2 RNA traces in the residential setting (5, 6). These results reinforce the utility of surface monitoring as a robust, cost-effective method for locating SARS-CoV-2 signals in the environment.

Our findings also corroborated SARS-CoV-2-associated changes in the microbiome that have been previously published. Rothia dentocariosa has been identified across different sample types in diverse settings, although the reasons for these associations remain unclear. We also note multiple sOTUs belonging to the genus Corynebacterium predictive as of a SARS-CoV-2 detection event, in contrast to the results of another study that found Corynebacterium significantly decreased in the oral microbiome of individuals with COVID-19 (11). We hypothesize that the *Corynebacterium* signal in this study might be evidence of human skin contamination of indoor surfaces through contact (21, 22), leading to SARS-CoV-2 deposition on surfaces. It has been established that the occupants of a room contribute to the environmental microbiota, but our findings are among the first to demonstrate that disease-associated changes in the microbiome are mirrored in the built environment.

## Supplementary Material

Reviewer comments

## References

[1] Fielding-Miller R, Karthikeyan S, Gaines T, Garfein RS, Salido R, Cantu V, Kohn L, Martin NK, Wijaya C, Flores M, Omaleki V, Majnoonian A, Gonzalez-Zuniga P, Nguyen M, Vo AV, Le T, Duong D, Hassani A, Dahl A, Tweeten S, Jepsen K, Henson B, Hakim A, Birmingham A, Mark AM, Nasamran CA, Rosenthal SB, Moshiri N, Fisch KM, Humphrey G, Farmer S, Tubb HM, Valles T, Morris J, Kang J, Khaleghi B, Young C, Akel AD, Eilert S, Eno J, Curewitz K, Laurent LC, Rosing T, SEARCH, Knight R. 2021. Wastewater and surface monitoring to detect COVID-19 in elementary school settings: the Safer at School Early Alert project. medRxiv 10.1101/2021.10.19.21265226. PMC9939935 36844610

[2] Jiang FC, Jiang XL, Wang ZG, Meng ZH, Shao SF, Anderson BD, Ma MJ. 2020. Detection of severe acute respiratory syndrome coronavirus 2 RNA on surfaces in quarantine rooms. Emerg Infect Dis 26:2162–2164. doi:10.3201/eid2609.201435. PMC7454114 32421495

[3] Zhou J, Otter JA, Price JR, Cimpeanu C, Meno Garcia D, Kinross J, Boshier PR, Mason S, Bolt F, Holmes AH, Barclay WS. 2021. Investigating severe acute respiratory syndrome coronavirus 2 (SARS-CoV-2) surface and air contamination in an acute healthcare setting during the peak of the coronavirus disease 2019 (COVID-19) pandemic in London. Clin Infect Dis 73:e1870–e1877. doi:10.1093/cid/ciaa905. 32634826 PMC7454437

[4] Ben-Shmuel A, Brosh-Nissimov T, Glinert I, Bar-David E, Sittner A, Poni R, Cohen R, Achdout H, Tamir H, Yahalom-Ronen Y, Politi B, Melamed S, Vitner E, Cherry L, Israeli O, Beth-Din A, Paran N, Israely T, Yitzhaki S, Levy H, Weiss S. 2020. Detection and infectivity potential of severe acute respiratory syndrome coronavirus 2 (SARS-CoV-2) environmental contamination in isolation units and quarantine facilities. Clin Microbiol Infect 26:1658–1662. doi:10.1016/j.cmi.2020.09.004. 32919072 PMC7481174

[5] Renninger N, Nastasi N, Bope A, Cochran SJ, Haines SR, Balasubrahmaniam N, Stuart K, Bivins A, Bibby K, Hull NM, Dannemiller KC. 2021. Indoor dust as a matrix for surveillance of COVID-19. mSystems 6:e01350-20. doi:10.1128/mSystems.01350-20. 33850045 PMC8547012

[6] Maestre JP, Jarma D, Yu JRF, Siegel JA, Horner SD, Kinney KA. 2021. Distribution of SARS-CoV-2 RNA signal in a home with COVID-19 positive occupants. Sci Total Environ 778:146201. doi:10.1016/j.scitotenv.2021.146201. 34030356 PMC7942153

[7] Wu S, Wang Y, Jin X, Tian J, Liu J, Mao Y. 2020. Environmental contamination by SARS-CoV-2 in a designated hospital for coronavirus disease 2019. Am J Infect Control 48:910–914. doi:10.1016/j.ajic.2020.05.003. 32407826 PMC7214329

[8] Marotz C, Belda-Ferre P, Ali F, Das P, Huang S, Cantrell K, Jiang L, Martino C, Diner RE, Rahman G, McDonald D, Armstrong G, Kodera S, Donato S, Ecklu-Mensah G, Gottel N, Salas Garcia MC, Chiang LY, Salido RA, Shaffer JP, Bryant MK, Sanders K, Humphrey G, Ackermann G, Haiminen N, Beck KL, Kim H-C, Carrieri AP, Parida L, Vázquez-Baeza Y, Torriani FJ, Knight R, Gilbert J, Sweeney DA, Allard SM. 2021. SARS-CoV-2 detection status associates with bacterial community composition in patients and the hospital environment. Microbiome 9:132. doi:10.1186/s40168-021-01083-0. 34103074 PMC8186369

[9] Dunn RR, Fierer N, Henley JB, Leff JW, Menninger HL. 2013. Home life: factors structuring the bacterial diversity found within and between homes. PLoS One 8:e64133. doi:10.1371/journal.pone.0064133. 23717552 PMC3661444

[10] Kembel SW, Jones E, Kline J, Northcutt D, Stenson J, Womack AM, Bohannan BJ, Brown GZ, Green JL. 2012. Architectural design influences the diversity and structure of the built environment microbiome. ISME J 6:1469–1479. doi:10.1038/ismej.2011.211. 22278670 PMC3400407

[11] Lax S, Smith DP, Hampton-Marcell J, Owens SM, Handley KM, Scott NM, Gibbons SM, Larsen P, Shogan BD, Weiss S, Metcalf JL, Ursell LK, Vazquez-Baeza Y, Van Treuren W, Hasan NA, Gibson MK, Colwell R, Dantas G, Knight R, Gilbert JA. 2014. Longitudinal analysis of microbial interaction between humans and the indoor environment. Science 345:1048–1052. doi:10.1126/science.1254529. 25170151 PMC4337996

[12] Wu Y, Cheng X, Jiang G, Tang H, Ming S, Tang L, Lu J, Guo C, Shan H, Huang X. 2021. Altered oral and gut microbiota and its association with SARS-CoV-2 viral load in COVID-19 patients during hospitalization. NPJ Biofilms Microbiomes 7:61. doi:10.1038/s41522-021-00232-5. 34294722 PMC8298611

[13] Gu S, Chen Y, Wu Z, Chen Y, Gao H, Lv L, Guo F, Zhang X, Luo R, Huang C, Lu H, Zheng B, Zhang J, Yan R, Zhang H, Jiang H, Xu Q, Guo J, Gong Y, Tang L, Li L. 2020. Alterations of the gut microbiota in patients with coronavirus disease 2019 or H1N1 influenza. Clin Infect Dis 71:2669–2678. doi:10.1093/cid/ciaa709. 32497191 PMC7314193

[14] Amir A, McDonald D, Navas-Molina JA, Kopylova E, Morton JT, Zech Xu Z, Kightley EP, Thompson LR, Hyde ER, Gonzalez A, Knight R. 2017. Deblur rapidly resolves single-nucleotide community sequence patterns. mSystems 2:e01350-20. doi:10.1128/mSystems.00191-16. PMC5340863 28289731

[15] Gonzalez A, Navas-Molina JA, Kosciolek T, McDonald D, Vázquez-Baeza Y, Ackermann G, DeReus J, Janssen S, Swafford AD, Orchanian SB, Sanders JG, Shorenstein J, Holste H, Petrus S, Robbins-Pianka A, Brislawn CJ, Wang M, Rideout JR, Bolyen E, Dillon M, Caporaso JG, Dorrestein PC, Knight R. 2018. Qiita: rapid, web-enabled microbiome meta-analysis. Nat Methods 15:796–798. doi:10.1038/s41592-018-0141-9. 30275573 PMC6235622

[16] Bolyen E, Rideout JR, Dillon MR, Bokulich NA, Abnet CC, Al-Ghalith GA, Alexander H, Alm EJ, Arumugam M, Asnicar F, Bai Y, Bisanz JE, Bittinger K, Brejnrod A, Brislawn CJ, Brown CT, Callahan BJ, Caraballo-Rodríguez AM, Chase J, Cope EK, Da Silva R, Diener C, Dorrestein PC, Douglas GM, Durall DM, Duvallet C, Edwardson CF, Ernst M, Estaki M, Fouquier J, Gauglitz JM, Gibbons SM, Gibson DL, Gonzalez A, Gorlick K, Guo J, Hillmann B, Holmes S, Holste H, Huttenhower C, Huttley GA, Janssen S, Jarmusch AK, Jiang L, Kaehler BD, Bin Kang K, Keefe CR, Keim P, Kelley ST, Knights D, et al. 2019. Reproducible, interactive, scalable and extensible microbiome data science using QIIME 2. Nat Biotechnol 37:852–857. doi:10.1038/s41587-019-0209-9. 31341288 PMC7015180

[17] Salido RA, Cantú VJ, Clark AE, Leibel SL, Foroughishafiei A, Saha A, Hakim A, Nouri A, Lastrella AL, Castro-Martínez A, Plascencia A, Kapadia BK, Xia B, Ruiz CA, Marotz CA, Maunder D, Lawrence ES, Smoot EW, Eisner E, Crescini ES, Kohn L, Vargas LF, Chacón M, Betty M, Machnicki M, Wu MY, Baer NA, Belda-Ferre P, De Hoff P, Seaver P, Ostrander RT, Tsai R, Sathe S, Aigner S, Morgan SC, Ngo TT, Barber T, Cheung W, Carlin AF, Yeo GW, Laurent LC, Fielding-Miller R, Knight R. 2021. Analysis of SARS-CoV-2 RNA persistence across indoor surface materials reveals best practices for environmental monitoring programs. mSystems 6:e0113621. doi:10.1128/mSystems.01136-21. 34726486 PMC8562474

[18] Minich JJ, Zhu Q, Janssen S, Hendrickson R, Amir A, Vetter R, Hyde J, Doty MM, Stillwell K, Benardini J, Kim JH, Allen EE, Venkateswaran K, Knight R. 2018. KatharoSeq enables high-throughput microbiome analysis from low-biomass samples. mSystems 3:e00218-17. doi:10.1128/mSystems.00218-17. 29577086 PMC5864415

[19] Morton JT, Marotz C, Washburne A, Silverman J, Zaramela LS, Edlund A, Zengler K, Knight R. 2019. Establishing microbial composition measurement standards with reference frames. Nat Commun 10:2719. doi:10.1038/s41467-019-10656-5. 31222023 PMC6586903

[20] Quinn TP, Erb I, Gloor G, Notredame C, Richardson MF, Crowley TM. 2019. A field guide for the compositional analysis of any-omics data. Gigascience 8:giz107. doi:10.1093/gigascience/giz107. 31544212 PMC6755255

[21] Byrd AL, Belkaid Y, Segre JA. 2018. The human skin microbiome. Nat Rev Microbiol 16:143–155. doi:10.1038/nrmicro.2017.157. 29332945

[22] Cantrell K, Fedarko MW, Rahman G, McDonald D, Yang Y, Zaw T, Gonzalez A, Janssen S, Estaki M, Haiminen N, Beck KL, Zhu Q, Sayyari E, Morton JT, Armstrong G, Tripathi A, Gauglitz JM, Marotz C, Matteson NL, Martino C, Sanders JG, Carrieri AP, Song SJ, Swafford AD, Dorrestein PC, Andersen KG, Parida L, Kim H-C, Vázquez-Baeza Y, Knight R. 2021. EMPress enables tree-guided, interactive, and exploratory analyses of multi-omic data sets. mSystems 6:e01216-20. doi:10.1128/mSystems.01216-20. 33727399 PMC8546999

