# Supplementary figures and images for: SARS-CoV-2 Distribution in Residential Housing Suggests Contact Deposition and Correlates with Rothia sp
Source: mSystems. 2022 May 16;7(3):e01411-21. doi: 10.1128/msystems.01411-21 (PMC9239251; doi:10.1128/msystems.01411-21)

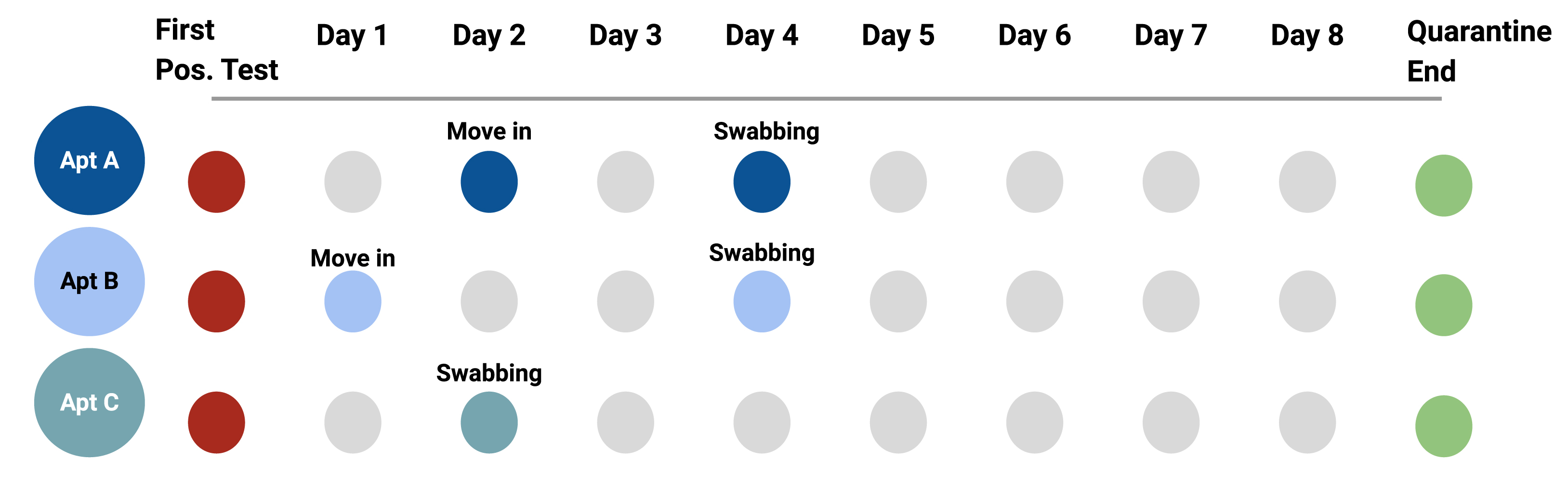

Supplement: FIG S1 [file msystems.01411-21-s0007.jpg]

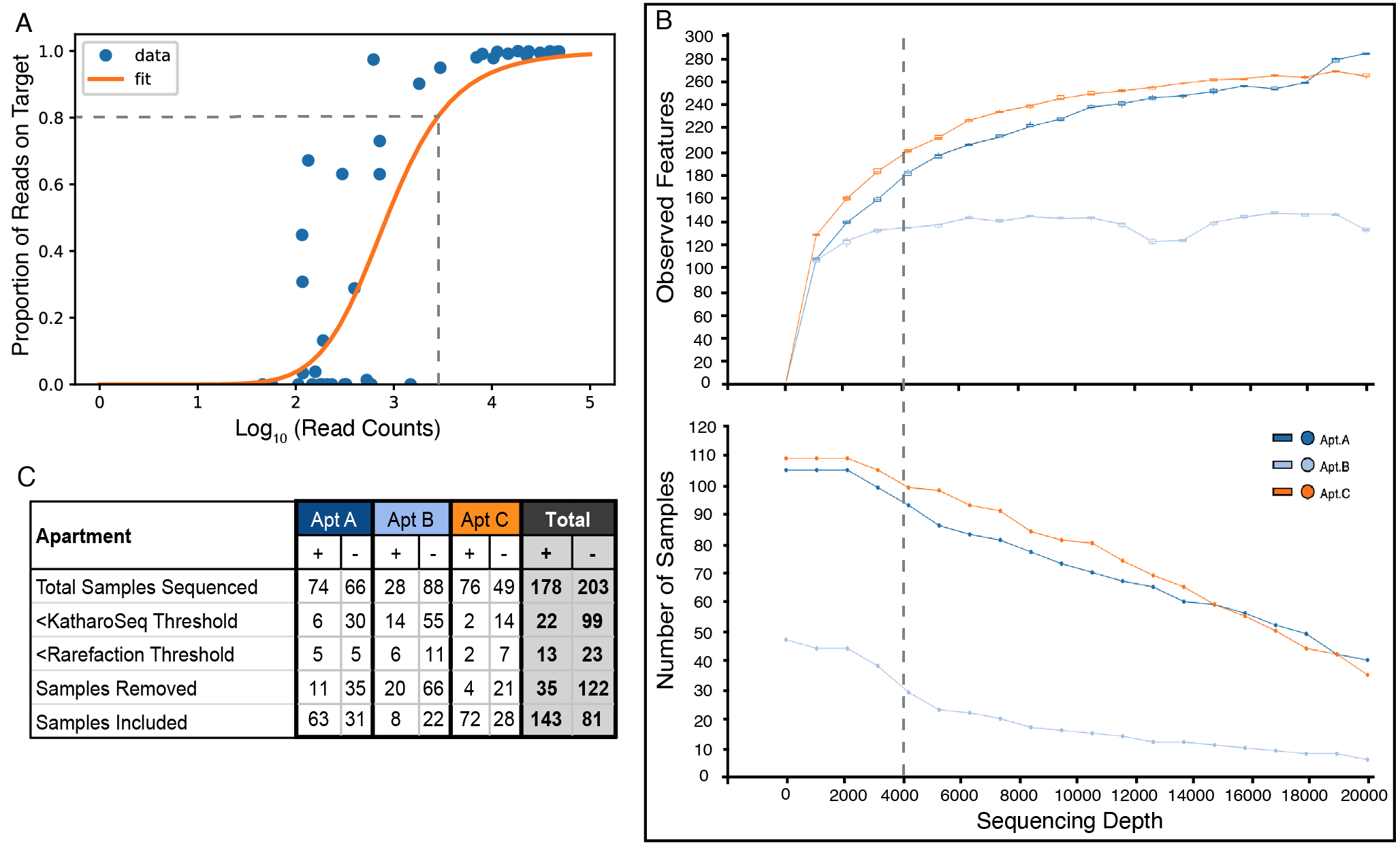

Supplement: FIG S2 [file msystems.01411-21-s0002.tif]

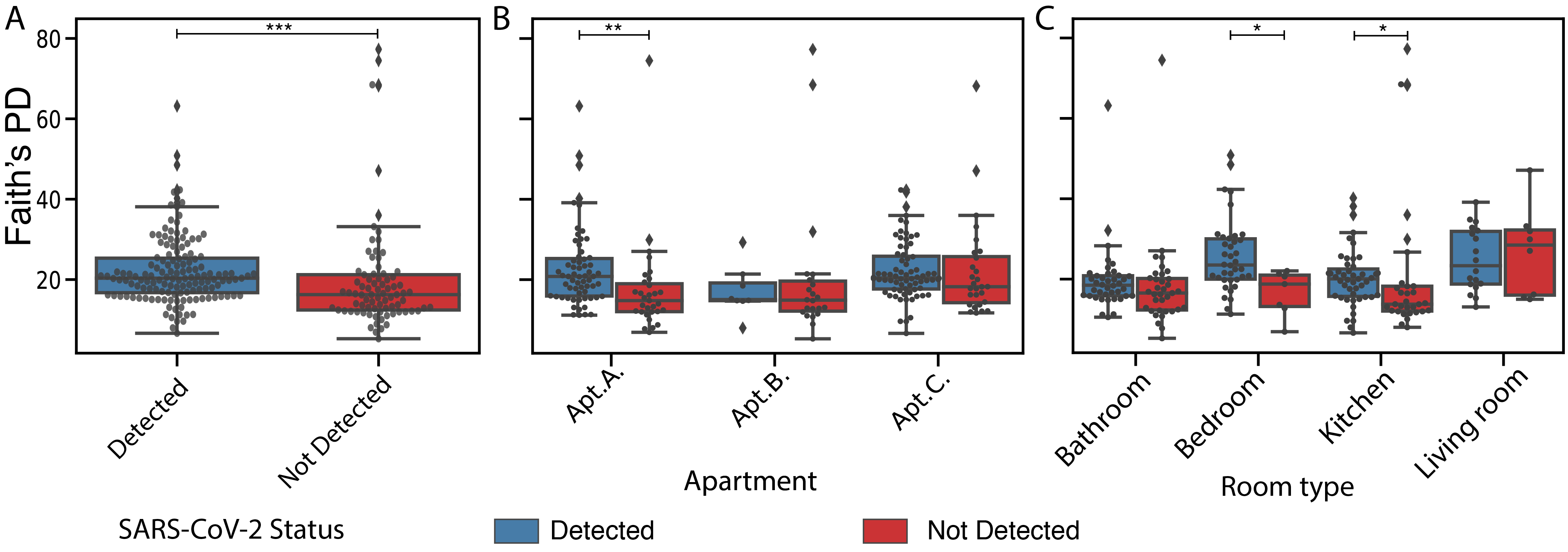

Supplement: FIG S3 [file msystems.01411-21-s0003.tif]

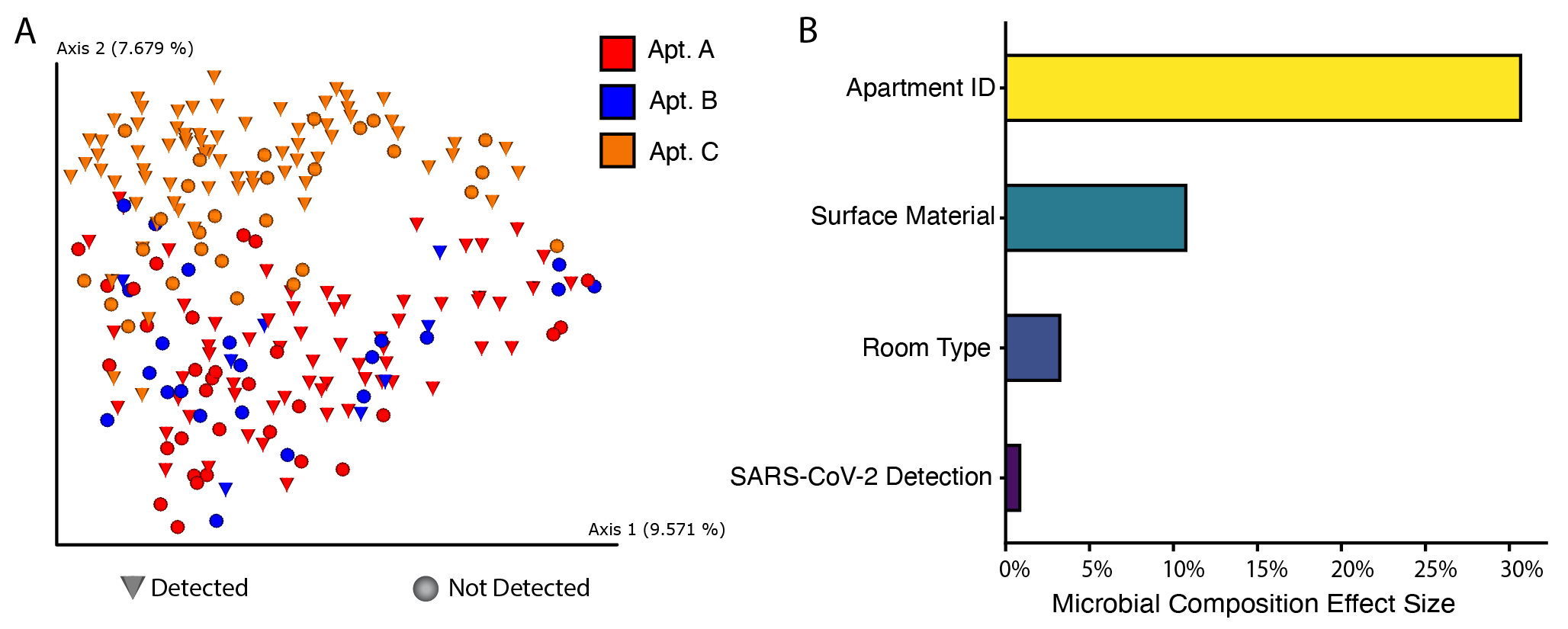

Supplement: FIG S4 [file msystems.01411-21-s0001.tif]
