# Supplementary material for: SARS-CoV-2 Distribution in Residential Housing Suggests Contact Deposition and Correlates with Rothia sp
Source: mSystems. 2022 May 16;7(3):e01411-21. doi: 10.1128/msystems.01411-21 (PMC9239251; doi:10.1128/msystems.01411-21)
Supplement: TEXT S1 [file msystems.01411-21-s0005.pdf]

Supplementary Materials and Methods.

Patient Recruitment:
In an effort to identify patients isolating in similar residential settings, the patient population focused on University California San Diego (UCSD) students isolating in the established, on-campus UCSD isolation dorm housing. Cases were identified through the UCSD Health system as COVID-19 positive outpatients with a positive anterior nares clinical RT-qPCR assay from the UCSD EXCITE (EXpedited COVID-19 IdenTification Environment) laboratory. Patients were recruited to the study via phone call, enrolled into an IRB-approved study (UCSD protocol 200477), and confirmed to be active UCSD students isolating in the isolation dorms. Three students were enrolled in the study: two of the students isolated in the on-campus isolation dorms, and the third isolated in their on-campus residence (graduate housing with similar architecture and design as the on-campus isolation dorms).

Surface Swabbing:
For each paired sample, two 1 mL sample collection tubes (ThermoFisher Scientific, 3740TS) were prepared. One tube contained 800 µL of 0.5% w/v sodium dodecyl sulfate (SDS) (Acros Organics, 230420025) in water and was used for detecting SARS-CoV-2, and the second tube contained 95% spectrophotometric-grade ethanol solution (Sigma-Aldrich #493511) which was designated for 16S sequencing. To recover genetic material from the surfaces, a prewashed cotton swab (Puritan, 806-WC) was pre-moistened with the ethanol solution and then used to vigorously swab the surface. The cotton end of the swab was then placed back into the sample collection tube and broken at the designated break point. The process was then immediately repeated on an adjacent site of the same surface with a flocculated tip swab (Affordable IHC Solutions) pre-moistened with the SDS solution, minimizing overlap between swabbed areas.

Viral Nucleic Acid Extraction and RT-qPCR:
Swabs stored in SDS were subjected to SARS-CoV-2 RT-qPCR detection following methods previously described (1). Briefly, 150µL of the SDS solution were extracted with Omega MagBind Viral DNA/RNA kit (Omega Bio-Tek, M6246) on Kingfisher Flex (ThermoFisher Scientific) instruments. Viral gene detection was performed using a miniaturized TaqPath™ COVID-19 Combo Kit (ThermoFisher Scientific, A47814) assay on a QuantStudio 7 Pro with a 384-well sample block (ThermoFisher Scientific).

Microbial Nucleic Acid Extraction:
Sample plating and extractions of all surface swabs were carried out in a biosafety cabinet Class II in a BSL2+ facility. Cotton tipped swabs suspended in 95% ethanol were plated into bead plates from 96 MagMAX™ Microbiome Ultra Nucleic Acid Isolation Kits (A42357 Thermo Fisher Scientific, USA). Following the KatharoSeq low biomass protocol (2), each sample processing plate included eight positive controls consisting of 10-fold serial dilutions of a microbial standard consisting of a gram negative *Paracoccus spp.* and gram positive *Bacillus subtilis* ranging from 5 to 50 million cells per extraction, and 3 negative controls (Blanks, sample-free lysis buffer). Nucleic acid extraction and purification was performed following

methods previously described (3). Briefly, samples were extracted in plates using the MagMAX™ Microbiome Ultra Nucleic Acid Isolation Kit (Applied Biosystems™), following manufacturer specifications, in KingFisher Flex™ robots (Thermo Fisher Scientific, USA), including a bead beating step in a TissueLyser II (Qiagen, Germany) at 30 Hz for 2 min.

16S Sequencing:
16S rRNA gene amplification was performed according to the Earth Microbiome Project protocol (4). Briefly, the V4 region of the 16S rRNA gene was targeted for amplification in a miniaturized reaction (5) using the 515f-806r primers with Golay error-correcting barcodes. Amplicons were pooled at equal volumes and the pool was purified with a QIAquick PCR purification kit (QIAGEN). The pooled libraries were sequenced on a MiSeq (Illumina) instrument with a MiSeq Reagent 300 cycle v2 Kit, with the appropriate sequencing primers. 16S sequencing data available in Qiita (Study ID: 13957) and EBI (ERP135867).

Estimating viral genomic equivalents:
To estimate viral genomic equivalents for each sample, we used published standard curves relating average Cqs from RT-qPCR to known SARS-CoV-2 viral particle concentrations (in GE's from digital droplet PCR) used to inoculate a variety of indoor surfaces (1). The equation used depended on which qualitative category the surface materials belonged to: rough (carpet, fabric) or smooth (e.g., acrylic, steel, glass, ceramic tile). The relationship between Cqs and GEs for rough materials is [GEs = -0.52 x (Avg Cq) + 39.90] while for smooth materials the equation used was [GEs = -0.77 x (Avg Cq) + 40.41].

Data processing:
16S sequences were demultiplexed, quality filtered, and denoised with Deblur (7) in Qiita (8) using default parameters. Resulting feature tables were processed using QIIME2 (9).

Katharoseq ***:***
In addition to the 381 samples that underwent 16S sequencing, three negative controls (blanks) and eight positive controls (a serially diluted bacterial stock, see *Microbial Nucleic Acid Extraction*) were included in each 96-well extraction plate. The positive controls were used to determine the threshold read count for which at least 80% of sequencing reads align to the positive controls (10).

Alpha Diversity:
To explore the relationship between microbial diversity and SARS-CoV-2 status, we compared the Faith's PD values of the microbiome samples between SARS-Cov-2 status groups (Detected/Not Detected) at different levels of sample subsetting (whole dataset, by apartment, and by room type). The relationship between microbial diversity (Faith's PD) and SARS-CoV-2 detection status was tested with a Mann-Whitney U test .2D Figures were made using matplotlib (11).

Beta Diversity:

We used the unweighted Unifrac phylogenetic distance (12-13) to explore how the microbial samples compare to each other. To quantify the effect size of different categorical variables on our data, redundancy analysis (RDA) was applied to the unweighted Unifrac principal coordinates. RDA estimates the contributions of individual and combined effects of multiple covariates using the *varpart* function in R to perform linear constrained ordination (14). 2D Figures were made using matplotlib (11) and EMPeror (15).

Differential Abundance:
To prepare the data for differential abundance, we filtered the unrarefied feature table to exclude features present in fewer than 10 samples and samples with depth less than 1000 reads. This resulted in a table of 258 samples and 1047 sOTUs. We performed multinomial regression using Songbird (16) (a compositionally aware multinomial regression method that operates on centered log-ratio feature coordinates) accounting for viral detection status, apartment, surface type, and indoor space classifier as covariates. We used 5000 epochs and a learning rate of 0.0001 as hyperparameters. Additionally, we specified a 3:1 split of training:testing samples for cross validation. To ensure that our model was not overfitting we fit a null regression model with no covariates using the same hyperparameters. Comparing the two models we found a positive pseudo-Q^2 value of 0.059, indicating that our regression model outperformed the null model.

Random Forest Classifier:
We performed machine learning analysis on the bacterial portion of the built environment surface microbiome from 16S sequencing to predict the samples' SARS-CoV-2 status from paired RT-qPCR detection results. Random forest classifiers were trained on the rarefied feature table and tested following a leave-one-site-out-cross-validation (LOSOCV) approach: the classifier was trained with samples from N-1 sites and its performance was tested in the remaining site using a precision-recall curve (Area Under the Precision Recall Curve (AUPRC), and Relative AUPRC). Classifiers were trained on sOTU-level features with tuned hyperparameters as 20-time repeated, LOSOCV, with sites resolved at the apartment_id (Fig. 2A) and room_type (Fig. 2B) levels using the R caret package(17). The classifiers' performance was evaluated with AUPRC based on the samples' SARS-CoV-2 status predictions of the holdout test site using the R PRROC package (18). The importance of each sOTU for the prediction performance of the classifiers was estimated by the built-in random forest scores in a 100-fold cross validation. We ranked the top 32 important features by their average ranking of importance scores across the 100 classification models. Relevant codebase for machine learning analysis is available at https://github.com/shihuang047/crossRanger and is based on random forest implementation from R ranger package (19).

Phylogenetic Tree visualization:
To identify phylogenetic clades important for the prediction of SARS-CoV-2 status from environmental surface samples, we visualized the top 32 important features identified by the random forest classifier and the ranked differentially abundant features between SARS-CoV-2 status groups from multinomial regression using EMPress (20). Briefly, a phylogenetic tree was built through SATé-enabled phylogenetic placement (SEPP) (21) of sOTU sequences into a

curated, reference tree (greengenes 13_8) (22); taxonomy of sOTUs, displayed as colored branches in the tree visualization, was assigned through a prefitted classifier (trained on greengenes 13_8 reference) (22). The leaves of the tree represent the intersection of the set of sOTUs in the rarefied and Katharoseq filtered feature table used in the microbiome diversity analysis, and the set of sOTUs evaluated for differential abundance with Songbird (total sOTUs = 1047). All of the aforementioned steps were performed with QIIME2 (9).

3D Mapping:
3D models were provided by UC San Diego's Housing, Dining, and Hospitality department. A circular target was placed on all swabbed locations in each apartment. 3D coordinates were picked following published methods (ref) (https://github.com/MolecularCartography/ili), and merged with viral load (in GEs) data for visualization. 3D models and merged data (coordinates and viral load) were visualized in ili (23).

References

1. Salido RA, Cantú VJ, Clark AE, Leibel SL, Foroughishafiei A, Saha A, Hakim A, Nouri A, Lastrella AL, Castro-Martínez A, Plascencia A, Kapadia BK, Xia B, Ruiz CA, Marotz CA, Maunder D, Lawrence ES, Smoot EW, Eisner E, Crescini ES, Kohn L, Vargas LF, Chacón M, Betty M, Machnicki M, Wu MY, Baer NA, Belda-Ferre P, Hoff P De, Seaver P, Ostrander RT, Tsai R, Sathe S, Aigner S, Morgan SC, Ngo TT, Barber T, Cheung W, Carlin AF, Yeo GW, Laurent LC, Fielding-Miller R, Knight R. 2021. Analysis of SARS-CoV-2 RNA Persistence across Indoor Surface Materials Reveals Best Practices for Environmental Monitoring Programs. mSystems https://doi.org/10.1128/MSYSTEMS.01136-21.

2. Minich JJ, Zhu Q, Janssen S, Hendrickson R, Amir A, Vetter R, Hyde J, Doty MM, Stillwell K, Benardini J, Kim JH, Allen EE, Venkateswaran K, Knight R. 2018. KatharoSeq Enables High-Throughput Microbiome Analysis from Low-Biomass Samples. mSystems 3.

3. Shaffer JP, Marotz C, Belda-Ferre P, Martino C, Wandro S, Estaki M, Salido RA, Carpenter CS, Zaramela LS, Minich JJ, Bryant M, Sanders K, Fraraccio S, Ackermann G, Humphrey G, Swafford AD, Miller-Montgomery S, Knight R. 2021. A comparison of DNA/RNA extraction protocols for high-throughput sequencing of microbial communities. Biotechniques btn-2020-0153.

4. Thompson LR, Sanders JG, McDonald D, Amir A, Ladau J, Locey KJ, et al. A communal catalogue reveals Earth's multiscale microbial diversity. Nature. 2017;551:457–63.

5. Minich JJ, Humphrey G, Benitez RAS, Sanders J, Swafford A, Allen EE, Knight R. 2018. High-Throughput Miniaturized 16S rRNA Amplicon Library Preparation Reduces Costs while Preserving Microbiome Integrity. mSystems 3.

6. Cruz GNF, Christoff AP, de Oliveira LFV. 2021. Equivolumetric Protocol Generates Library Sizes Proportional to Total Microbial Load in 16S Amplicon Sequencing. Front Microbiol 12.

7. Amir A, McDonald D, Navas-Molina JA, Kopylova E, Morton JT, Zech Xu Z, Kightley EP, Thompson LR, Hyde ER, Gonzalez A, Knight R. 2017. Deblur Rapidly Resolves Single-Nucleotide Community Sequence Patterns. mSystems 2.

8. Gonzalez A, Navas-Molina JA, Kosciolek T, McDonald D, Vázquez-Baeza Y, Ackermann G, DeReus J, Janssen S, Swafford AD, Orchanian SB, Sanders JG, Shorenstein J, Holste H, Petrus S, Robbins-Pianka A, Brislawn CJ, Wang M, Rideout JR, Bolyen E, Dillon M, Caporaso JG, Dorrestein PC, Knight R. 2018. Qiita: rapid, web-enabled microbiome meta-analysis. Nat Methods 15:796–798.

9. Bolyen E, Rideout JR, Dillon MR, Bokulich NA, Abnet CC, Al-Ghalith GA, Alexander H, Alm EJ, Arumugam M, Asnicar F, Bai Y, Bisanz JE, Bittinger K, Brejnrod A, Brislawn CJ, Brown CT, Callahan BJ, Caraballo-Rodríguez AM, Chase J, Cope EK, Da Silva R, Diener C, Dorrestein PC, Douglas GM, Durall DM, Duvallet C, Edwardson CF, Ernst M, Estaki M, Fouquier J, Gauglitz JM, Gibbons SM, Gibson DL, Gonzalez A, Gorlick K, Guo J, Hillmann B, Holmes S, Holste H, Huttenhower C, Huttley GA, Janssen S, Jarmusch AK, Jiang L, Kaehler BD, Kang K Bin, Keefe CR, Keim P, Kelley ST, Knights D, Koester I, Kosciolek T, Kreps J, Langille MGI, Lee J, Ley R, Liu Y-X, Loftfield E, Lozupone C, Maher M, Marotz C, Martin BD, McDonald D, McIver LJ, Melnik A V., Metcalf JL, Morgan SC, Morton JT, Naimey AT, Navas-Molina JA, Nothias LF, Orchanian SB, Pearson T, Peoples SL, Petras D, Preuss ML, Pruesse E, Rasmussen LB, Rivers A, Robeson MS, Rosenthal P, Segata N, Shaffer M, Shiffer A, Sinha R, Song SJ, Spear JR, Swafford AD, Thompson LR, Torres PJ, Trinh P, Tripathi A, Turnbaugh PJ, Ul-Hasan S, van der Hooft JJJ, Vargas F, Vázquez-Baeza Y, Vogtmann E, von Hippel M, Walters W, Wan Y, Wang M, Warren J, Weber KC, Williamson CHD, Willis AD, Xu ZZ, Zaneveld JR, Zhang Y, Zhu Q, Knight R, Caporaso JG. 2019. Reproducible, interactive, scalable and extensible microbiome data science using QIIME 2. Nat Biotechnol 37:852–857.

10. Minich JJ, Zhu Q, Janssen S, Hendrickson R, Amir A, Vetter R, Hyde J, Doty MM, Stillwell K, Benardini J, Kim JH, Allen EE, Venkateswaran K, Knight R. 2018. KatharoSeq Enables High-Throughput Microbiome Analysis from Low-Biomass Samples. mSystems 3.

11. Hunter JD. 2007. Matplotlib: A 2D graphics environment. Comput Sci Eng 9:90–95.

12. Lozupone C, Lladser ME, Knights D, Stombaugh J, Knight R. 2011. UniFrac: an effective distance metric for microbial community comparison. ISME J 5:169.

13. McDonald D, Vázquez-Baeza Y, Koslicki D, McClelland J, Reeve N, Xu Z, Gonzalez A, Knight R. 2018. Striped UniFrac: enabling microbiome analysis at unprecedented scale. Nat Methods 2018 1511 15:847–848.

14. Falony G, Joossens M, Vieira-Silva S, Wang J, Darzi Y, Faust K, Kurilshikov A, Bonder MJ, Valles-Colomer M, Vandeputte D, Tito RY, Chaffron S, Rymenans L, Verspecht C, Sutter L De, Lima-Mendez G, D'hoe K, Jonckheere K, Homola D, Garcia R, Tigchelaar EF, Eeckhaudt L, Fu J, Henckaerts L, Zhernakova A, Wijmenga C, Raes J. 2016. Population-level analysis of gut microbiome variation. Science (80-) 352:560–564.

15. Vázquez-Baeza Y, Pirrung M, Gonzalez A, Knight R. 2013. EMPeror: a tool for visualizing high-throughput microbial community data. Gigascience 2:16.

16. Morton JT, Marotz C, Washburne A, Silverman J, Zaramela LS, Edlund A, Zengler K, Knight R. 2019. Establishing microbial composition measurement standards with reference frames. Nat Commun 10:2719.

17. Kuhn M. 2008. Building Predictive Models in R Using the caret Package. J Stat Softw 28:1–26.

18. Keilwagen J, Grosse I, Grau J. 2014. Area under Precision-Recall Curves for Weighted and Unweighted Data. PLoS One 9:e92209.

19. Wright MN, Ziegler A. 2017. ranger: A Fast Implementation of Random Forests for High Dimensional Data in C++ and R. J Stat Softw 77:1–17.

20. Cantrell K, Fedarko MW, Rahman G, McDonald D, Yang Y, Zaw T, Gonzalez A, Janssen S, Estaki M, Haiminen N, Beck KL, Zhu Q, Sayyari E, Morton JT, Armstrong G, Tripathi A, Gauglitz JM, Marotz C, Matteson NL, Martino C, Sanders JG, Carrieri AP, Song SJ, Swafford AD, Dorrestein PC, Andersen KG, Parida L, Kim H-C, Vázquez-Baeza Y, Knight R. 2021. EMPress Enables Tree-Guided, Interactive, and Exploratory Analyses of Multi-omic Data Sets. mSystems 6.

21. Janssen S, McDonald D, Gonzalez A, Navas-Molina JA, Jiang L, Xu ZZ, Winker K, Kado DM, Orwoll E, Manary M, Mirarab S, Knight R. 2018. Phylogenetic Placement of Exact Amplicon Sequences Improves Associations with Clinical Information. mSystems 3.

22. McDonald D, Price MN, Goodrich J, Nawrocki EP, Desantis TZ, Probst A, Andersen GL, Knight R, Hugenholtz P. 2012. An improved Greengenes taxonomy with explicit ranks for ecological and evolutionary analyses of bacteria and archaea. ISME J 6:610.

23. Protsyuk I, Melnik A V., Nothias LF, Rappez L, Phapale P, Aksenov AA, Bouslimani A, Ryazanov S, Dorrestein PC, Alexandrov T. 2017. 3D molecular cartography using LC–MS facilitated by Optimus and 'ili software. Nat Protoc 2017 131 13:134–154.
